# Calcitriol Induces Paraoxonase 1 Expression in HepG2 Cells: Possible Involvement of VDR-Dependent and Alternative Pathways

**DOI:** 10.3390/ijms26167948

**Published:** 2025-08-18

**Authors:** Fidel Navarro-García, Aurora E. Rojas-García, Gabriela Ávila-Villarreal, Sergio Hidalgo-Figueroa, Briscia S. Barrón-Vivanco, Cyndia A. González-Arias, Yael Y. Bernal-Hernández, José F. Herrera-Moreno, Guillermo Elizondo, José L. Medina-Franco, Irma M. Medina-Díaz

**Affiliations:** 1 Posgrado en Ciencias Biológico Agropecuarias, Universidad Autónoma de Nayarit, Km. 9 Carretera Tepic-Compostela, Xalisco 63780, Nayarit, Mexico; 19000822@uan.edu.mx; 2 Laboratorio de Contaminación y Toxicología Ambiental, Secretaría de Investigación y Posgrado, Universidad Autónoma de Nayarit, Tepic 63000, Nayarit, Mexico; erojas@uan.edu.mx (A.E.R.-G.); bbarron@uan.edu.mx (B.S.B.-V.); cyndia.gonzalez@uan.edu.mx (C.A.G.-A.); yael.bernal@uan.edu.mx (Y.Y.B.-H.); francisco.herrera@uan.edu.mx (J.F.H.-M.); 3 Unidad Académica de Ciencias Químico Biológicas y Farmacéuticas, Universidad Autónoma de Nayarit, Tepic 63000, Nayarit, Mexico; gaby.avila@uan.edu.mx; 4 Centro Nayarita de Innovación y Transferencia de Tecnología “Unidad especializada en I+D+i en Calidad de Alimentos y Productos Naturales”, Universidad Autónoma de Nayarit, Tepic 63000, Nayarit, Mexico; 5 CONACYT-División de Biología Molecular, Instituto Potosino de Investigación Científica y Tecnológica A.C., San Luis Potosí 78000, San Luis Potosí, Mexico; sergio.hidalgo@ipicyt.edu.mx; 6 Secretaría de Ciencia, Humanidades, Tecnología e Innovación (SECIHTI), Padrón de Investigadoras e Investigadores por México, Mexico City 03940, Mexico; 7 Departamento de Biología Celular, CINVESTAV-IPN, Mexico City 07360, Mexico; gazuela@cinvestav.mx; 8 Grupo de Investigación DIFACQUIM, Departamento de Farmacia, Facultad de Química, Universidad Nacional Autónoma de México, Mexico City 04510, Mexico; medinajl@unam.mx

**Keywords:** paraoxonase 1, calcitriol, gene expression, enzymatic activity, vitamin D receptor, pregnenolone X receptor

## Abstract

Paraoxonase 1 (PON1) is an antioxidant enzyme that plays physio-pathological roles. Prior in silico analysis revealed the presence of response elements of the nuclear receptor superfamily in the *PON1* promoter, comparable to glucocorticoid receptors (GR), the vitamin D receptor (VDR), and the pregnenolone X receptor (PXR). The aim of this study was to evaluate the effects of 1α,25-dihydroxyvitamin D_3_, a ligand specific to VDR, on the expression and activity of PON1 in hepatocarcinoma cells (HepG2 cells). PON1 activities (arylesterase/AREase and lactonase/LACase) were determined by spectrophotometry. Quantitative real-time PCR was used to evaluate the effect of VDR and PXR on the mRNA levels of *PON1* and *CYP3A4* genes. Molecular models and dynamics simulations were built using specialized software. Treatments with 1α,25-dyhydroxyvitamin D_3_ (calcitriol), its active hormonal form, resulted in an induction of *PON1* mRNA and AREase activity compared to control cultures. These results suggest that calcitriol plays a role in the regulation of *PON1* transcription and provide evidence that this hormone increases PON1 levels in HepG2 cells. In addition, the molecular modeling suggests that calcitriol enhances PON1 activity and this increase could be caused by direct interaction on the PON1 protein. This study shows the effects of calcitriol on *PON1* expression, proposing a new molecular mechanism for the transcriptional regulation of *PON1* through a process linked to VDR activation and direct interaction of calcitriol on the PON1 protein.

## 1. Introduction

Paraoxonase 1 (PON1) belongs to a family of three enzymes (PON1, PON2, and PON3), playing a key role in the hydrolyzation of organophosphorus compounds, such as the active metabolite of parathion, paraoxon [1]. All of them have lactonase activity, but only PON1 strongly hydrolyzes paraoxon [1]. PON1 is a calcium-dependent enzyme, mainly synthesized in the liver and secreted into plasma in a complex with high-density lipoproteins (HDL) [2]. During the last two decades, there have been implications of the role of PON1 in the development of various chronic diseases [3,4], such as cardiovascular diseases (CVD). This has been reported, and research groups have highlighted the antioxidant property of PON1 in its capability to hydrolyze oxidized lipids [5,6].

Several factors including single nucleotide polymorphisms, exercise, exposure to environmental pollutants, drug consumption, and dietary and harmful habits modulate the expression and activity of PON1 [7,8]. Given the implications of this enzyme regarding health status, the transcriptional regulation of the *PON1* gene is under study in order to increase its messenger RNA (mRNA) and resulting protein product [9]. In silico analysis revealed the presence of response elements (RE) with 95% homology to members of the nuclear receptor (NR) superfamily in the *PON1* promoter, such as the glucocorticoid receptors (GR) alpha and beta, the vitamin D receptor (VDR), the pregnenolone X receptor (PXR), and peroxisome proliferator-activated receptor (PPAR) alpha [10]. Some fibrates have also been shown to increase the promoter activity of *PON1* [11]. However, some statins have opposite effects as they decrease mRNA levels and PON1 activity, mediated by PPAR [12]. In vitro studies demonstrated that quercetin, resveratrol, and aspirin induce *PON1* expression through the aryl hydrocarbon receptor (AhR), supporting the suggestions made by Gouédard et al. [13] that the *PON1* promoter contains a nonclassical xenobiotic response element (GCGGG). Ponce-Ruíz et al. [10] showed that treatments on HepG2 cells with dexamethasone (GR ligand) and TCDD (AhR ligand) increased the mRNA levels of *PON1*, and the activation of GR by dexamethasone results in *PON1* gene induction.

However, no functional studies have yet been conducted focusing on elucidating the role of PXR and VDR in regulating *PON1* expression. PXR mainly regulates the expression of genes involved in drug biotransformation and functions as a biosensor by means of cholesterol homeostatic regulation and bile acid metabolism [12], and the VDR regulates the transcription of genes associated with calcium absorption, ossification, cell cycle, and biotransformation of xenobiotics [14]. Given that the promoter region of *PON1* exhibited response elements for PXR, VDR, and retinoid X receptor (RXR) [10], as well as the essential role as an antioxidant molecule in lipid metabolism and in the development of chronic diseases such as CVD, it is consequently important to investigate the mechanisms involved in the regulation of *PON1* expression.

The aim of this study was to evaluate the effects of calcitriol, specific ligand to VDR, on the expression and activity of PON1 in HepG2 cells.

## 2. Results

### 2.1. CYP3A4 and PON1 mRNA Expression Levels

The mRNA expression of CYP3A4 was used as a positive control for PXR and VDR.

**Figure 1:**
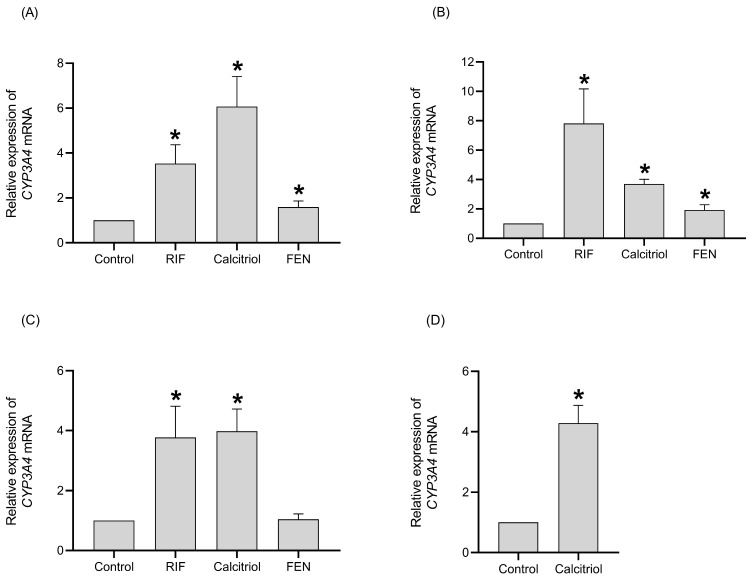
Effect of RIF, calcitriol, and FEN on *CYP3A4* mRNA levels in HepG2 cells after 24 h (**A**), 48 h (**B**), 72 h (**C**), and 96 h (**D**) of treatment. HepG2 cells were treated with 0.05% DMSO (control), 10 µM RIF, 0.25 µM calcitriol, and 250 µM FEN. The relative expression of *CYP3A4* mRNA was determined by real-time PCR and normalized to the *GAPDH* mRNA levels. The results are expressed as the mean ± standard deviation (SD) of three independent experiments in duplicate. The p values were calculated using the Mann–Whitney U test (* *p* < 0.05 vs. control).

Treatment with RIF (10 µM), calcitriol (0.25 µM), and FEN (250 µM) increased the expression of *CYP3A4* mRNA at 24, 48, 72, and 96 h (Figure 1). An increase of 3.5- and 3.7-fold were observed in *CYP3A4* mRNA at 24 and 72 h for RIF treatment (Figure 1A,C), and maximal induction was observed at 48 h (7.8-fold) (Figure 1B). Also, calcitriol treatments increased *CYP3A4* mRNA levels in all evaluated times, and maximal induction was observed at 24 h (6.0-fold) compared to the control (Figure 1A), whereas treatments with FEN increased *CYP3A4* mRNA after 24 and 48 h (1.6- and 1.9-fold) compared to the controls (Figure 1A,B).

**Figure 2:**
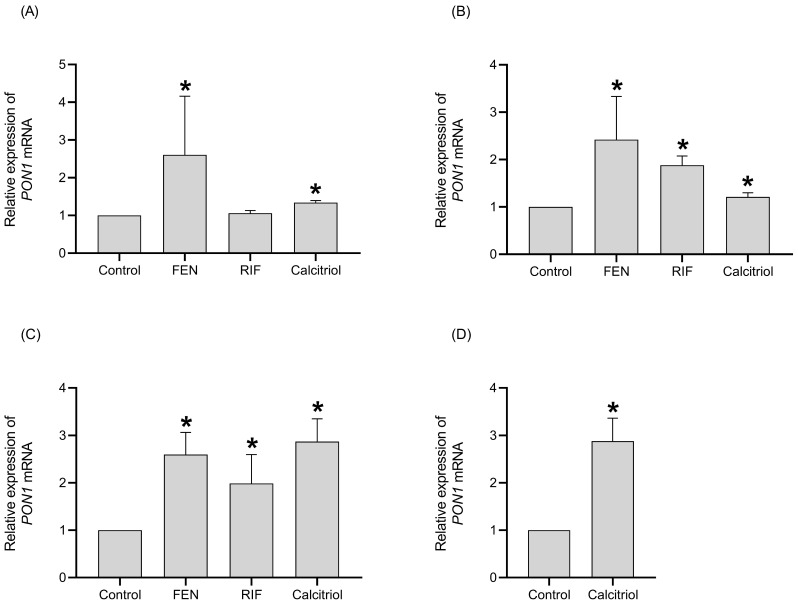
Effect of FEN, RIF, and calcitriol on *PON1* mRNA levels in HepG2 cells after 24 h (**A**), 48 h (**B**), 72 h (**C**), and 96 h (**D**) of treatment. HepG2 cells were treated with 0.05% DMSO (control), 250 µM FEN, 10 µM RIF, and 0.25 µM calcitriol. The relative expression of *PON1* mRNA was determined by real-time PCR and normalized to the *18S* mRNA levels. The results are expressed as the mean ± standard deviation (SD) of three independent experiments in duplicate. The *p* values were calculated using the Mann–Whitney U test (* *p* < 0.05 vs. control).

Regarding the effect of PXR and VDR ligands on *PON1* mRNA levels, the results showed that RIF (10 µM) induces the *PON1* mRNA levels (2-fold) at 48 and 72 h of treatment compared with the control (Figure 2B,C). Also, the treatments with calcitriol (0.25 µM) increased *PON1* mRNA levels from 24 h of treatment (Figure 2A), showing a maximal fold induction (2.8 times higher than controls) at 72 h (Figure 2C), which was maintained until 96 h (Figure 2D).

**Figure 3:**
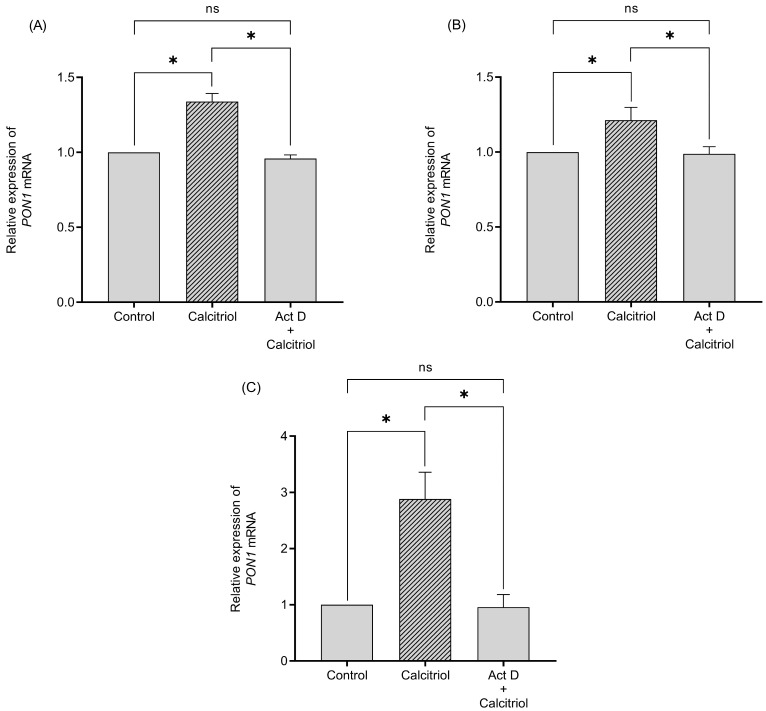
Inhibition of *PON1* expression by actinomycin D (Act D) after 24 h (**A**), 48 h (**B**), and 72 h (**C**) of treatment. HepG2 cells were pretreated with 10 µg/mL Act D for 10 min followed by the addition of calcitriol. The *PON1* mRNA levels were determined and normalized to the *18S* mRNA levels. The results are expressed as the mean ± standard deviation (SD) of three independent experiments in duplicate. The *p* values were calculated using the Mann–Whitney U test (* *p* < 0.05). ns: not statistically significant.

Regarding the effect of calcitriol on *PON1* mRNA levels, the results demonstrated that the induction of *PON1 mRNA* synthesis by calcitriol was decreased by pretreatment with Act D (Figure 3). This evaluation was only performed for calcitriol, as the induction of *PON1* at the transcriptional level by RIF and FEN was previously demonstrated by our research group [15].

Based on the data shown, this is the first time that the induction of *PON1* by calcitriol involves active transcription and the first suggestion that VDR (calcitriol) ligands could be involved in such an induction.

### 2.2. PON1 Activity

**Figure 4:**
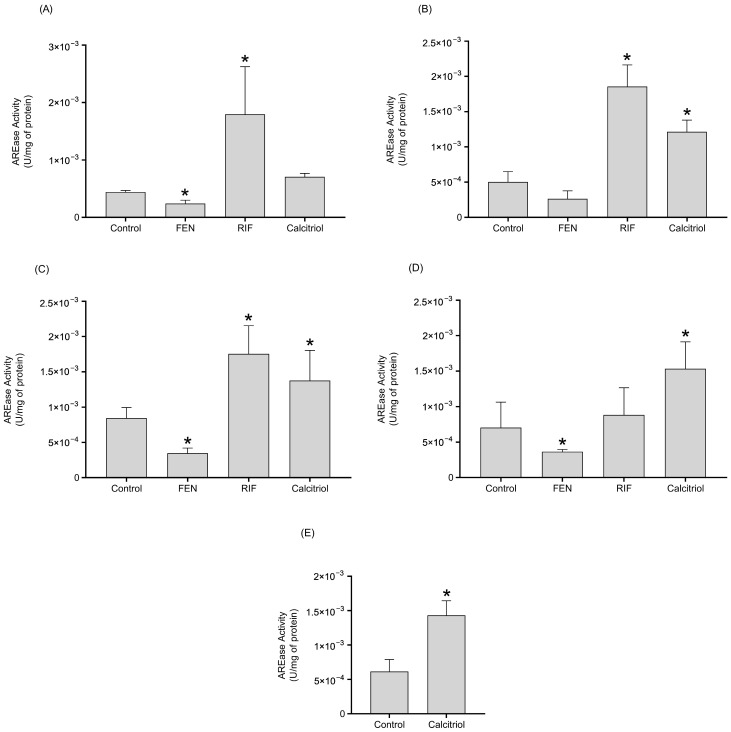
Effect of FEN, RIF, and calcitriol on the AREase activity of PON1 in HepG2 cells after 24 h (**A**), 48 h (**B**), 72 h (**C**), 96 h (**D**), and 120 h (**E**, only calcitriol) of treatment. HepG2 cells were treated with 0.05% DMSO (control), 250 µM FEN, 10 µM RIF, and 0.25 µM calcitriol. PON1 activity toward phenyl acetate was measured. The results are expressed as the mean ± standard deviation of three independent (SD) experiments by duplicate. The *p* values were calculated using the Mann–Whitney U test (* *p* < 0.05 vs. control).

**Figure 5:**
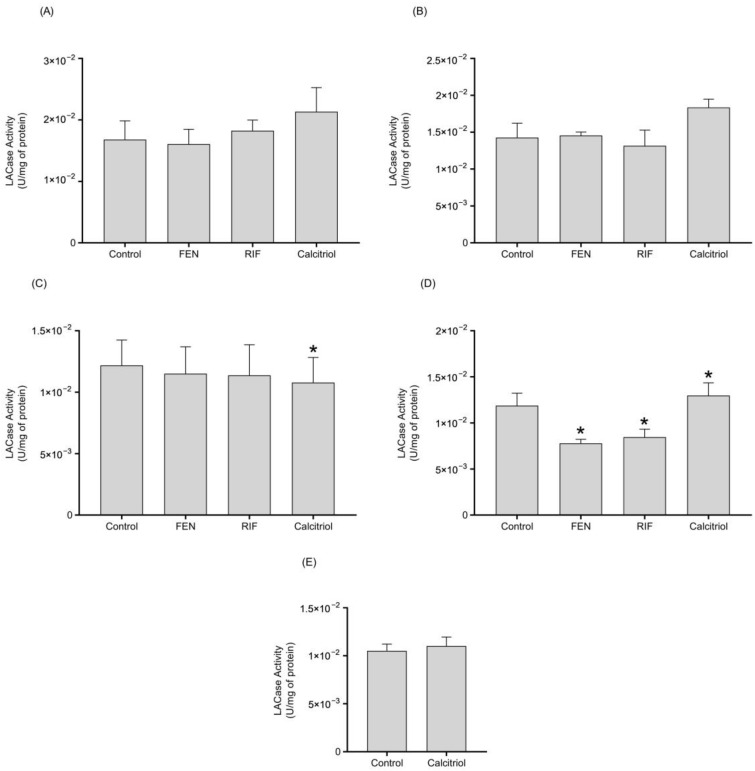
Effect of FEN, RIF, and calcitriol on the LACase activity of PON1 in HepG2 cells after 24 h (**A**), 48 h (**B**), 72 h (**C**), 96 h (**D**), and 120 h (**E**, only calcitriol) of treatment. HepG2 cells were treated with 0.05% DMSO (control), 250 µM FEN, 10 µM RIF, and 0.25 µM calcitriol. The PON1 activity towards dihydrocoumarin was measured. The results are expressed as the mean ± standard deviation of three independent experiments by duplicate. The *p* values were calculated using the Mann–Whitney’ U test (* *p* < 0.05 vs. control).

The results revealed that the increases in *PON1* mRNA expression by RIF and calcitriol are able to spike AREase PON1 activity (Figure 4). Also, only the calcitriol treatment increased the LACase activity at 72 h (Figure 5).

The treatment with RIF (10 µM) increased AREase activity at 24, 48, and 72 h of treatment (Figure 4), while LACase activity decreased after 96 h of treatment (Figure 5). Also, calcitriol increased PON1 AREase activity after 48 h of treatment (Figure 4). Similarly to the behavior of LACase activity with RIF, the treatment with calcitriol decreased LACase activity at 72 h, and then there was observed an increase at 96 h in comparison to the control (Figure 5). In terms of mRNA and the activity of PON1, the data suggest the possible upregulation of the *PON1* gene by PXR and VDR.

### 2.3. Molecular Docking

**Figure 6:**
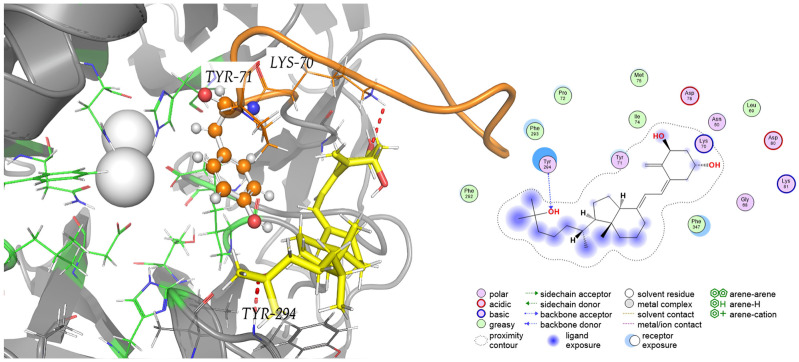
PON1–calcitriol complex with 2D interaction diagram. The green residues (sticks) indicate the binding site, the orange residue shows TYR71 (ball and sticks), yellow indicates the ligand, and red dashed lines denote the hydrogen bonds.

Docking study of calcitriol was carried out with an X-ray structure of PON1. The X-ray crystal structures were modified by remodeling the flexible loop KYPGIMSFDPDK (residues 70–81), as described in the Methods section. The model with the best DOPE score was used in molecular docking. According to the literature, this loop contains a functionally critical Tyr71 and certain mutants on flexible loop showed markedly different effects on different activities [16,17]. In this study, after selecting the optimal structure, we assessed the binding mode of the calcitriol into the active site covering the flexible loop. The docking model shown in Figure 6 indicates that calcitriol has the potential to occupy an area between the open loop and the surface and that its binding is mediated by hydrogen bonds from Lys70 and Tyr294, with a calculated binding energy of −7.5 Kcal/mol. Based on the docking model, we hypothesized that the mechanism governing the enhancement effect of the activity enzyme was the blockage in this area. To further investigate the potential mechanism of inhibition of calcitriol considering the flexibility of the system, the generated PON–calcitriol complex obtained with docking (Figure 6) was used as a starting point for MD simulations.

### 2.4. Molecular Dynamics

**Figure 7:**
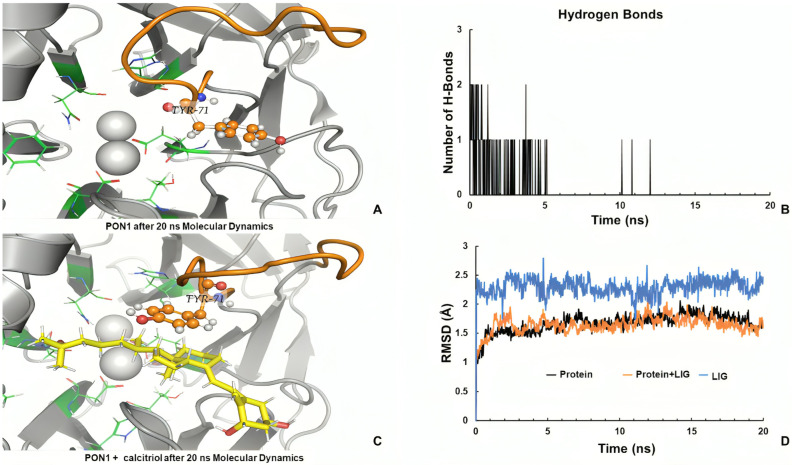
Three-dimensional representation of PON1 in (**A**) the absence of ligand and (**C**) in the presence of ligand. (**B**) Hydrogen bond interactions formed during MD simulation and (**D**) Root Mean Square Deviation (RMSD) plot of the systems and calcitriol during the 20 ns MD simulations. The green residues (sticks) indicate the binding site, the orange residue shows TYR71 (ball and sticks), yellow indicates the ligand.

Based on preliminary results, we decided to study the performance of calcitriol in the PON1–calcitriol complex. The initial PON1 modified structure was submitted to MD simulations in absence of the ligand. Figure 7A shows the Tyr71 orientation, which is conserved outside of enzymatic cavity after 20 ns. Otherwise, Tyr71 was oriented to cleft of the active site in the presence of calcitriol, indicating that it is more exposed to the recognition of substrates.

Although calcitriol was losing contact with residues of the binding site during the MD simulations, after 10 ns (Figure 7B), the new molecular conformation was constant during the last ns of simulation (Figure 7C); it also allowed loop stability with a favorable Tyr71 orientation, which is important for substrate recognition [16,17]. The RMSD of calcitriol, complex, and protein were stable during the MD simulations (Figure 7D). These results support the experimental evidence that calcitriol increases the PON1 activity.

## 3. Discussion

The regulation of PON1 by internal and external factors was reported earlier [18,19]. However, to date, the molecular mechanisms involved in this process remain unknown. PON1 plays a crucial role as an antioxidant to HDL and in the prevention of various chronic diseases [20]. This study is the first time that calcitriol has been shown to increase the expression and activity of PON1 in HepG2 cells. These results indicate that calcitriol could be an enhancement of PON1 activity, an increase that could be the result of direct interaction of calcitriol on the PON1 protein.

Previous studies carried out analysis of the promoter region of *PON1* and found response elements for PXR, GR, VDR, and RXR [10,21]. However, the effect of VDR ligands has not been thoroughly examined on the expression of *PON1*. Thus, certain effects of xenobiotics and endogenous molecules are due to the interaction of the nuclear receptors [22]. Our results showed that treatments with FEN (250 µM), RIF (10 µM), and calcitriol (0.25 µM) induce *PON1* mRNA levels at all of the times evaluated. These results denote the active role of PXR (RIF) and VDR (calcitriol) ligands, which could be involved in the induction of *PON1*. To the best of our knowledge, this is the first study to demonstrate an increase in the transcription of *PON1* by ligands to VDR. However, the mechanism of action by which ligands to VDR and PXR increase or modulate the expression of *PON1* remains unknown. Previously, our research group reported the induction of *PON1* expression by dexamethasone and rifampicin, suggesting that de novo synthesis of mRNA is the principal mechanism of induction (proven in the Act D treatments) [10]. Our data suggest that the same mechanism could occur with calcitriol treatments, given that pretreatment with Act D significantly decreased the levels of *PON1* mRNA synthesis at all of the times assessed, indicating that the induction of *PON1* by calcitriol involves active transcription, as the *PON1* gene promoter possesses a response element for the VDR. Regarding the activity of PON1 by RIF treatment, the increase in AREase activity could be explained by the increase in the mRNA *PON1* levels. Likewise, RIF is also capable of inducing the expression of interleukin 6 (IL6) inflammatory environments [23]. In this sense, the decrease in the activity of LACase after 96 h of treatment with RIF could be the result, at least in part, of the inverse relationship that prevails between *PON1* and inflammatory cytokines [24].

Although we observed a marked increase in *PON1* mRNA levels after treatment with FEN and RIF, this did not translate into an increase in lactonase activity, likely because functional PON1 synthesis is subject to post-transcriptional and post-translational controls (translation efficiency, folding, glycosylation, secretion, stability, and turnover rate) [25]. In addition, the amount of active enzyme available in the supernatant is influenced by dose, duration of treatment, and conditions in the culture medium [9]. In this study, FEN and RIF may not have generated the conditions or stability over time to raise lactonase activity, unlike calcitriol at 96 h. Nevertheless, direct quantification of PON1 concentration would have better elucidated this discrepancy between the induced mRNA levels and lactonase activity.

The information regarding the influence of vitamin D, specifically of the hormonal active form (calcitriol), on the regulation of the gene expression of *PON1* is limited. The effects of vitamins on *PON1* expression and activity had been centered on vitamins C and E [10]; recent studies have reported the influence of B-complex vitamins on animal models and human populations in terms of PON1 status [26,27]. Also, some studies failed to find a relationship between vitamin intake and PON1 phenotypes. The daily intake of 400 IU of vitamin D during the last trimester in Serbian pregnant women demonstrated no changes in PON1 activity using paraoxon and diazoxon as substrates [28]. A pilot study conducted on Greek adolescents with overweight and obesity reported that 12 weeks of daily ingestion of 2000 IU of vitamin D did not have any effect on AREase or PONase activities [29]. However, Chehsmazar et al. [30] observed that the daily oral supplementation of 2000 IU of vitamin D over 8 weeks increased PON1 plasmatic concentration in Iranian overweight and obese adults (58.5 ± 25 vs. 80 ± 25 ng/mL).

As mentioned previously, there is an inverse relationship between the expression and activity of PON1 and proinflammatory cytokines. In this regard, the repression or silencing of inflammation-related genes in calcitriol-treated HepG2 cultures could be associated with the induction of the *PON1* mRNA levels and AREase activity reported in this study. Under normal conditions, the nuclear factor kappa-light-chain-enhancer of activated B cells (NF-κB) is sequestered by inhibitors of κB (IκB) that suppress its transcriptional activity. Proinflammatory processes indirectly activate NF-κB by the degradation of IκB following phosphorylation mediated by IκB kinase (IKK). Free NF-κB binds to response elements in the promoters of interleukins, increasing transcription [31]. It has been reported that calcitriol inhibits NF-κB through the induction of IκB by VDR transactivation, inhibiting NF-κB transcriptional activity and repressing the expression of interleukins [32]. Additionally, calcitriol treatment induces IL-10 [33], an anti-inflammatory cytokine that inhibits IKK activity and NF-κB DNA binding [34]. These findings support our theory in which increases in expression and activity of PON1 are not only due to the interaction of activated VDR to its response element in the *PON1* promoter but also involves a more complex mechanism of interaction with other transcription factors and proteins such as NK-κB, IκB, IKK, and IL-10. Crosstalk along the route of the VDR on the regulation of NF-κB, the anti-inflammatory cytokines, and the xenobiotics metabolizing enzyme system may occur during xenobiotic metabolism and oxidative stress, indicating the possible involvement of this receptor in the regulation of the *PON1* gene.

Moreover, recent evidence suggests that calcitriol may exert biological effects independently of VDR by acting through alternative nuclear receptors, including retinoic acid-related orphan receptors (RORs) and AhR, thereby expanding the range of its regulatory capabilities [35]. In addition, certain vitamin D and lumisterol metabolites have been shown to directly inhibit enzymes involved in viral replication, including those of SARS-CoV-2, without requiring VDR activation [36]. These findings support the possibility that calcitriol and its derivatives could regulate gene expression and enzymatic activity through receptor-independent mechanisms. This further strengthens our hypothesis that the regulation of PON1 by calcitriol may involve alternative pathways beyond classical VDR signaling.

The calcitriol concentrations studied in this work to activate the VDR are not cytotoxic [15]. The wide availability of this hormone, either endogenously or through the diet and the subsequent activation of the VDR, opens a new perspective in the search for new mechanisms of action in the regulation of the expression of antioxidant genes such as *PON1*.

To our knowledge, this work establishes, for the first time, that calcitriol could be an enhancer of PON1 activity and this increase could be through the direct interaction of the calcitriol protein on PON1. As we show in Figure 6, Tyr71 was oriented to the slit of the active site in the presence of calcitriol, which indicates that it is more exposed to the recognition of substrates. Notably, PON1 features a flexible loop spanning residues 70–81 that governs access to the catalytic pocket by adopting closed and open conformations [25,37]. In our docking model, calcitriol binds to a peripheral pocket adjacent to this loop, stabilized by hydrogen bonds with Lys70 and Tyr294, instead of residing within the catalytic pocket itself. Molecular dynamics simulations demonstrate that the Tyr71 residue remains out in place, preserving the state of the open loop leaving the catalytic cleft unobstructed. This allosteric stabilization of the open conformation probably facilitates substrate entry, providing a structural basis for calcitriol potentiation in PON1 activity.

Moreover, Tyr71 is not only critical for substrate access but also for anchoring PON1 to HDL. Structural and functional findings have shown that Tyr71 sits within a flexible loop that penetrates the lipid monolayer of HDL particles, engaging directly with membrane lipids; oxidation of this residue abolishes enzyme binding and activity on HDL [38]. Adequate circulating concentrations of vitamin D enhance both PON1 expression and HDL levels, thereby increasing the amount of functional PON1 associated with HDL [38,39]. Through its anti-inflammatory and antioxidant actions, calcitriol may preserve Tyr71 from oxidative inactivation and support hepatic PON1 secretion [38], thereby enhancing PON1’s antioxidant activity in its HDL-associated form.

In relation to the possible transcriptional regulation of the *PON1* gene through VDR, the results obtained show that calcitriol was able to increase the levels of *PON1* mRNA at all times evaluated. A maximum induction of 2.8 times was observed with respect to the control at 72 h of treatment, which was maintained until 96 h. These results suggest that the putative site identified in the promoter of the *PON1* gene by in silico analysis, with a similarity of 95% with respect to the consensus sequence of VDR [10], might be mediating the expression of *PON1*.

Since the necessary concentrations of calcitriol to show a favorable effect at the transcriptional level are not cytotoxic, the use of calcitriol certainly poses a new perspective in the search for mechanisms of action and regulation of antioxidant genes with perspectives for the search of therapeutic objectives that help to counteract diseases related to a deficit in the levels of expression of *PON1* and other genes, through the transactivation of VDR.

Our results were shown in the HepG2 cell line, one of the most widely used in vitro models to study gene expression and enzyme activity [40], including the enzymatic pathways mediated by nuclear receptors such as VDR and PXR [10,15,41,42]. However, HepG2 has been reported to have an incomplete metabolic capability when compared with primary hepatocytes, with lower levels of some phase I and II enzymes [40]. Nevertheless, HepG2 cells are genetically stable, easy to culture, and exhibit inter-batch reproducibility, making them a good platform for mechanistic nuclear signaling studies, dose response evaluation, and genetic manipulations like transfections or editing [43,44].

Therefore, in future studies, it would be appropriate to incorporate primary human hepatocytes as a complementary model to more rigorously compare the results obtained in HepG2 cells. In addition, more studies are needed to evaluate the interaction of the activated VDR on the response element in the *PON1* gene promoter to demonstrate that the induction of *PON1* by calcitriol is through VDR. Also, longitudinal studies are needed to evaluate how nutritional intake of calcitriol could influence PON1 activity.

## 4. Material and Methods

### 4.1. Materials and Reagents

Fenofibrate (FEN), rifampicin (RIF) 1α,25-dihydroxyvitamin D_3_ (calcitriol), 3-(4,5-dimethylthiazol-2-yl)-2,5-diphenyltetrazolium bromide (MTT), dithiothreitol (DTT), actinomycin D (Act D), phenylacetate (PA), and dihydrocoumarin (DHC) were purchased from Sigma-Aldrich (St. Louis, MO, USA). HepG2 cells (HB-8065; Lot number: 70032516). Eagle’s minimum essential medium (EMEM), along with L-glutamine, fetal bovine serum (FBS), and dimethyl sulfoxide (DMSO) were obtained from the American Type Culture Collection (ATCC) (Manassas, VA, USA). Nonessential amino acids and antibiotics–antimycotics were purchased from Gibco (Grand Island, NY, USA). TRIzol reagent, Oligo(dT), and SuperScript^TM^ III RNase H reverse transcriptase were purchased from Invitrogen Technologies (Carlsbad, CA, USA). PCR reagents, probes, and TaqMan Universal PCR Master Mix were obtained from Applied Biosystems, Inc. (Foster City, CA, USA). Glycerol was purchased from USB Corporation (Cleveland, OH, USA). Phenylmethylsulphonyl fluoride (PMSF) and the modified Lowry protein assay kit were obtained from Thermo Scientific (Meridian, Rockford, IL, USA). Tris–HCl was purchased from Promega Corporation (Madison, WI, USA), and CaCl_2_ was purchased from Mallinckrodt Baker, S.A. de C.V. (Mexico City, Mexico).

### 4.2. Cell Culture and Treatment

HepG2 cells [Hep G2 [HEPG2]—HB-8065] were cultured in EMEM with L-glutamine supplemented with 10% (*v*/*v*) inactivated FBS, 1% non-essential amino acids, and 1% antibiotics–antimycotics [10]. The cell cultures were maintained in 75 mm flasks in an incubator at 5% CO_2_ and 37 °C atmospheric temperature. Passages 4–9 were used in the experiments conducted.

The cells were plated at a density of 1 × 10^6^ cells/mL and allowed to attach for 24 h before the treatments. Treatments were performed in 12-well plates containing 1 × 10^6^ cells per well. HepG2 cells were treated with RIF 10 μM for 72 h, and with 0.25 μM calcitriol 96 h. The concentration and time of calcitriol treatments were chosen according to a previous study [15], in which VDR activation was validated via RT-PCR (CYP3A4 mRNA), Western blot (CYP3A4 protein), and viability assay, demonstrating effective gene regulation without impacting cell viability. Treatment with FEN 250 μM was used as the positive control of *PON1* expression. The ligand concentration was renewed every 24 h. Cell cultures treated with DMSO ≤ 0.05% were utilized as a control. The treatments were carried out in three independent experiments in duplicate.

### 4.3. Cell Viability by Determination of Metabolic Capacity

The MTT assay was used to measure cellular metabolic activity as an indicator of cell viability. Metabolic activity was evaluated according to Mosmann [45]. Briefly, HepG2 cells were harvested, counted in a Neubauer chamber, and seeded, with 5 × 10^5^ cells per well in a 96-well culture plate (Ref: 3595; Costar^®^, Corning Incorporated, Clinton, TN, USA). Cells were treated with RIF 10 μM and FEN 250 μM for 24, 48, 72, and 96 h. After treatment, 20 μL MTT (5 mg/mL in PBS 1X) was added to each well, and cells were incubated for 3 h at 37 °C in darkness. Then, 100 μL of the solubilization solution (0.1 N HCl in anhydrous isopropanol) was added to dissolve the formazan crystals, and the optical density was determined at 570 nm in an ELISA-plate reader (μQuant Bio Tek, Winooski, VT, USA). Cell viability was expressed as a percentage of absorbance with respect to solvent control absorbance (DMSO ≤ 0.05%). Neither viability nor metabolic activity were affected by RIF, calcitriol, and FEN treatments at 72 h treatment time when compared with controls. However, at 96 h of RIF and FEN treatments, a decreased in cell viability of more than 70% was observed. 

### 4.4. Isolation of Total RNA

Total RNA from HepG2 cell cultures was isolated by homogenizing cells in TRIzol reagent according to manufacturer’s instructions (Invitrogen Technologies).

### 4.5. cDNA Synthesis

cDNA was prepared from total RNA using SuperScript^TM^ III, according to the manufacturer’s instructions (Invitrogen Technologies).

### 4.6. Analysis of CYP3A4 and PON1 mRNA Expression

#### Quantitative Real-Time Polymerase Chain Reaction (qRT-PCR)

Real-time PCR assays of the transcripts were performed using gene-specific fluorescent labeled probes in a StepOne^TM^ sequence detector (Applied Biosystems). The following probe sequences were used: *PON1* (5′-CCATGTTGTAGCAAACCCTCAAGCT-3′) (Hs00166557_m1) and cytochrome P450 3A4 (*CYP3A4*) (5′-ATTTTGTCCTACCATAAGGGCTTTT-3′) (Hs00604506_m1). The mRNA expression of *CYP3A4* was utilized as a positive control for PXR and VDR [15]. Glyceraldehyde-3-phosphate dehydrogenase (*GAPDH*; Hs99999905_m1) and *18S* ribosomal RNA (rRNA; Hs99999901_s1) were validated for stable expression in HepG2 cells and were used for normalization of the mRNA data. The results were analyzed using the comparative threshold cycle method [46].

### 4.7. PON1 Enzymatic Activity

Cell cultures were treated 24 h longer than gene expression assays to obtain the maximal possible translation of mRNA.

PON1 arylesterase (AREase) activity was measured using phenylacetate as a substrate as previously described by Deakin et al. [47] and Ponce-Ruíz et al. [10]. After treatments, cells were washed three times with 1 mL of phosphate-buffered saline (PBS) and then incubated for 10 min with 900 μL of appropriate buffer (10 Mm Tris–HCl [pH 8.0], 1 mM CaCl_2_) and 100 μL of 10 mM phenyl acetate as a substrate. The supernatant was obtained and the change in absorbance at 270 nm was monitored for 5 min at 1 min intervals by spectrophotometry (Spectronic Genesys 10 Bio; ThermoScientific^®^, Waltham, MA, USA). The molar extinction coefficient of phenol (1310 M^−1^ cm^−1^) was used to calculate the hydrolysis of phenylacetate. A unit of ARE activity was equivalent to 1 μmol of hydrolyzed phenylacetate/min/mL.

Lactonase (LACase) activity was determined according to Billecke et al. [48] with some modifications and using dihydrocoumarin (DHC) as substrate. Briefly, HepG2 cells were washed three times with sterile 1X PBS. Cells were incubated in a reaction mixture composed of 990 μL of assay buffer (40 mM Tris-HCl, 1 mM CaCl_2,_ pH 8.0) and 10 μL of 100 mM DHC. OD at 270 nm was measured every 30 s for 3 min at 25 °C in a Spectronic GENESYS^TM^ 10 spectrophotometer from Bio-Thermo Scientific (Waltham, MA, USA) to determine the extent of DHC hydrolysis. Freshly prepared reaction mixture was used as a blank. LACase activity was determined using the molar extinction coefficient of 3-(*o*-hydroxyphenyl) propionic acid (ε = 1295 M^−1^ cm^−1^) and was expressed in U/mg of total protein.

Total protein extraction was quantified directly after enzymatic activity assessment using the modified Lowry protein assay kit.

We also performed molecular modeling to evaluate, at the molecular level, the potential interaction of calcitriol with PON1 as a substrate.

### 4.8. Molecular Modeling

#### 4.8.1. Structure Editing of Flexible Loop

The three-dimensional coordinates of PON1 were retrieved from the Protein Data Bank (PDB) (www.wwpdb.org) [49] with the accession code PDB ID: 3SRG and a resolution of 2.19 Å. The flexible active site loop (comprising residues 70–81) [50] were remodeled in Chimera 1.16 [51,52] in the Model/Refine loop section, which was made with the loop modeling protocol (DOPE protocol) using Modeller 10.0 [53].

#### 4.8.2. Molecular Docking Study of Calcitriol

The chemical structure of calcitriol was constructed in MOE 2022.02 [54]. Calcitriol was docked with the remodeled 3SRG structure in order to find the recognition mode against the receptor. The MOE 2022.02 software was used to protonate PON1 and calcitriol. The docking of calcitriol was assessed using AutoDock Vina 1.2.0 [55,56]. The grid box was fixed at the following coordinates: X = −13.38; Y = −22.529; and Z = 32.935 (centered covering the active site and flexible loop). The grid dimensions were 40 × 40 × 30 points, separated by 1 Å.

#### 4.8.3. Molecular Dynamics Simulation of PON1–Calcitriol Complex

Molecular dynamics (MD) simulations of 20 ns were conducted to explore the stability and dynamic behavior of the PON1–calcitriol complex. Simulations were performed using the NAnoscale Molecular Dynamics (NAMD) software package version 2.14 (https://www.ks.uiuc.edu/Development/Download/download.cgi?PackageName=NAMD, accessed 18 January 2023) [57] with the CHARMM36 force field.

Initially, the best-scoring pose obtained from molecular docking was protonated and submitted to the LigParGen web server (http://zarbi.chem.yale.edu/ligpargen/, accessed on 18 January 2023) [58,59,60] to generate the topology (.rtf), parameter (.prm), and coordinate (.pdb) files for the ligand.

The resulting PON1–calcitriol complex was embedded in a cubic simulation box, solvated with TIP3P water molecules, and neutralized by adding chloride (Cl^−^) ions. Energy minimization was carried out for 2 ps at 273.15 K, applying harmonic restraints to the protein backbone to relax solvent interactions and relieve steric clashes.

Following minimization, the system was subjected to 20 ns of unrestrained MD simulation under isothermal–isobaric (NPT) conditions at 300 K and 1 atm pressure, using periodic boundary conditions and a 2 fs integration time step.

Visual Molecular Dynamics (VMD) software 1.9.3 (https://www.ks.uiuc.edu/Research/vmd/, accessed 18 January 2023) [61] was used as both the interface and analysis tool throughout the simulation process.

### 4.9. Statistical Analysis

The data were analyzed and presented as mean ± standard deviation (SD) to summarize the central tendency and variability of the results. To determine differences between groups, the Mann–Whitney U test, a non-parametric test suitable for comparing two independent samples, was employed due to the non-normal distribution of the data. This test was chosen as it does not assume homogeneity of variance or normality, making it appropriate for small sample sizes or skewed data. To assess the significance of the differences observed between the groups, *p*-values were calculated, with a threshold of *p* < 0.05 considered statistically significant. The analysis was conducted using STATA software version 14 (Stata Corporation, College Station, TX, USA).

## Data Availability

The data that support the findings of this study are available from the corresponding author upon reasonable request.

## References

[1] Furlong C.E., Marsillach J., Jarvik G.P., Costa L.G. (2016). Paraoxonases-1, -2 and -3: What are their functions?. Chem. Interact..

[2] Précourt L.-P., Marcil V., Ntimbane T., Taha R., Lavoie J.-C., Delvin E., Seidman E.G., Beaulieu J.-F., Levy E. (2012). Antioxidative properties of paraoxonase 2 in intestinal epithelial cells. Am. J. Physiol. Liver Physiol..

[3] Androutsopoulos V.P., Kanavouras K., Tsatsakis A.M. (2011). Role of paraoxonase 1 (PON1) in organophosphate metabolism: Implications in neurodegenerative diseases. Toxicol. Appl. Pharmacol..

[4] Paul K.C., Sinsheimer J.S., Cockburn M., Bronstein J.M., Bordelon Y., Ritz B. (2017). Organophosphate pesticides and PON1 L55M in Parkinson’s disease progression. Environ. Int..

[5] Kresanov P., Vasankari T., Ahotupa M., Kaikkonen J., Hutri-Kähönen N., Juonala M., Kähönen M., Lehtimäki T., Viikari J., Raitakari O.T. (2015). Paraoxonase-1 and oxidized lipoprotein lipids. The Cardiovascular Risk in Young Finns Study. Atherosclerosis.

[6] González F.E.M., Ponce-Ruíz N., Rojas-García A.E., Bernal-Hernández Y.Y., Mackness M., Ponce-Gallegos J., Cardoso-Saldaña G., Jorge-Galarza E., Torres-Tamayo M., Medina-Díaz I.M. (2019). PON1 concentration and high-density lipoprotein characteristics as cardiovascular biomarkers. Arch. Med. Sci. Atheroscler. Dis..

[7] Otocka-Kmiecik A., Orlowska-Majdak M. (2009). The role of genetic (PON1 polymorphism) and environmental factors, especially physical activity, in antioxidant function of paraoxonase. Postepy Hig. Med. Dosw..

[8] Longo A., Veiga G.B., Cousen M.I.S., Karpinski C., Schneider A., Weber B., Bertoldi E.G., Borges L.R., Bertacco R.T.A. (2021). Factors associated to serum paraoxonase 1 activity in patients with cardiovascular disease. Arq. Bras. Endocrinol. Metabol..

[9] Fuhrman B. (2012). Regulation of Hepatic Paraoxonase-1 Expression. J. Lipids.

[10] Ponce-Ruiz N., Rojas-García A., Barrón-Vivanco B., Elizondo G., Bernal-Hernández Y., Mejía-García A., Medina-Díaz I. (2015). Transcriptional regulation of human paraoxonase 1 by PXR and GR in human hepatoma cells. Toxicol. Vitr..

[11] Gouédard C., Koum-Besson N., Barouki R., Morel Y. (2003). Opposite regulation of the human paraoxonase-1 Gene PON-1 by fenofibrate and statins. Mol. Pharmacol..

[12] Wallace B.D., Betts L., Talmage G., Pollet R.M., Holman N.S., Redinbo M.R. (2013). Structural and functional analysis of the human nuclear xenobiotic receptor PXR in complex with RXRα. J. Mol. Biol..

[13] Gouédard C., Barouki R., Morel Y. (2004). Dietary polyphenols increase paraoxonase 1 gene expression by an aryl hydrocarbon receptor-dependent mechanism. Mol. Cell. Biol..

[14] Carlberg C., Campbell M.J. (2013). Vitamin D receptor signaling mechanisms: Integrated actions of a well-defined transcription factor. Steroids.

[15] Elizondo G., Medina-Díaz I.M. (2003). Induction of CYP3A4 by 1α,25-dyhydroxyvitamin D_3_ in HepG2 cells. Life Sci..

[16] Ben-David M., Elias M., Filippi J.-J., Duñach E., Silman I., Sussman J.L., Tawfik D.S. (2012). Catalytic Versatility and Backups in Enzyme Active Sites: The Case of Serum Paraoxonase 1. J. Mol. Biol..

[17] Sierra-Campos E., Valdez-Solana M., Avitia-Domínguez C., Campos-Almazán M., Flores-Molina I., García-Arenas G., Téllez-Valencia A. (2020). Effects of *Moringa oleifera* Leaf Extract on Diabetes-Induced Alterations in Paraoxonase 1 and Catalase in Rats Analyzed through Progress Kinetic and Blind Docking. Antioxidants.

[18] Costa L.G., Giordano G., Furlong C.E. (2011). Pharmacological and dietary modulators of paraoxonase 1 (PON1) activity and expression: The hunt goes on. Biochem. Pharmacol..

[19] Rajkovic M.G., Rumora L., Barisic K. (2011). The paraoxonase 1, 2 and 3 in humans. Biochem. Medica.

[20] Grzegorzewska A.E., Adamska P., Iwańczyk-Skalska E., Ostromecka K., Niepolski L., Marcinkowski W., Mostowska A., Warchoł W., Żaba C., Jagodziński P.P. (2021). Paraoxonase 1 concerning dyslipidaemia, cardiovascular diseases, and mortality in haemodialysis patients. Sci. Rep..

[21] Schrader C., Rimbach G. (2011). Determinants of Paraoxonase 1 status: Genes, drugs and nutrition. Curr. Med. Chem..

[22] Tao L.J., Seo D.E., Jackson B., Ivanova N.B., Santori F.R. (2020). Nuclear Hormone Receptors and Their Ligands: Metabolites in Control of Transcription. Cells.

[23] Ziglam H., Daniels I., Finch R. (2004). Immunomodulating Activity of Rifampicin. J. Chemother..

[24] Han C.Y., Chiba T., Campbell J.S., Fausto N., Chaisson M., Orasanu G., Plutzky J., Chait A. (2006). Reciprocal and coordinate regulation of serum amyloid A vs. apolipoprotein A-I and paraoxonase-1 by inflammation in murine hepatocytes. Arter. Thromb. Vasc. Biol..

[25] Taler-Verčič A., Goličnik M., Bavec A. (2020). The Structure and Function of Paraoxonase-1 and Its Comparison to Paraoxonase-2 and -3. Molecules.

[26] Ponce-Ruiz N., Murillo-González F.E., Rojas-García A.E., Bernal-Hernández Y.Y., Mackness M., Ponce-Gallegos J., Barrón-Vivanco B.S., Hernández-Ochoa I., González-Arias C.A., Ortega-Cervantes L. (2020). Phenotypes and concentration of PON1 in cardiovascular disease: The role of nutrient intake. Nutr. Metab. Cardiovasc. Dis..

[27] Sarandol E., Tas S., Serdar Z., Dirican M. (2020). Effects of thiamine treatment on oxidative stress in experimental diabetes. Bratisl. Med. J..

[28] Ardalić D., Stefanović A., Kotur-Stevuljević J., Vujović A., Spasić S., Spasojević-Kaliomanvska V., Jelić-Ivanović Z., Mandić-Marković V., Miković Z., Cerović N. (2014). The influence of maternal smoking habits before pregnancy and antioxidative supplementation during pregnancy on oxidative stress status in a non-complicated pregnancy. Adv. Clin. Exp. Med..

[29] Makariou S.E., Challa A., Siomou E., Tellis C., Tselepis A., Elisaf M., Liberopoulos E. (2020). Vitamin D status and cardiometabolic risk factors in Greek adolescents with obesity—The effect of vitamin D supplementation: A pilot study. Arch. Med. Sci. Atheroscler. Dis..

[30] Chehsmazar E., Zarrati M., Yazdani B., Razmpoosh E., Hosseini A.F., Shidfar F. (2021). The effect of vitamin D supplementation on serum concentrations of dehydroepiandrosterone, paraoxonase 1, apolipoproteins, free fatty acid and insulin in vitamin D deficient obese and overweight individuals under a low-calorie diet program: A randomized controlled trial. Nutr. Food Sci..

[31] Liu T., Zhang L., Joo D., Sun S.-C. (2017). NF-κB signaling in inflammation. Signal Transduct. Target. Ther..

[32] Krishnan A.V., Feldman D. (2011). Mechanisms of the anti-cancer and anti-inflammatory actions of vitamin D. Annu. Rev. Pharmacol. Toxicol..

[33] Matilainen J.M., Husso T., Toropainen S., Seuter S., Turunen M.P., Gynther P., Ylä-Herttuala S., Carlberg C., Väisänen S. (2010). Primary effect of 1α,25(OH)_2_D_3_ on IL-10 expression in monocytes is short-term down-regulation. Biochim. Biophys. Acta (BBA) Mol. Cell Res..

[34] Schottelius A.J.G., Mayo M.W., Sartor R.B., Baldwin A.S. (1999). Interleukin-10 signaling blocks inhibitor of κB kinase activity and nuclear factor κB DNA binding. J. Biol. Chem..

[35] Slominski A.T., Kim T.-K., Janjetovic Z., Slominski R.M., Li W., Jetten A.M., Indra A.K., Mason R.S., Tuckey R.C. (2024). Biological Effects of CYP11A1-Derived Vitamin D and Lumisterol Metabolites in the Skin. J. Investig. Dermatol..

[36] Qayyum S., Mohammad T., Slominski R.M., Hassan I., Tuckey R.C., Raman C., Slominski A.T. (2021). Vitamin D and lumisterol novel metabolites can inhibit SARS-CoV-2 replication machinery enzymes. Am. J. Physiol. Metab..

[37] Lewoń-Mrozek D., Kurzynoga J., Jędrzejewski P., Kędzierska K., Partyka A., Kuriata-Kordek M., Ściskalska M. (2024). Molecular Structure of Paraoxonase-1 and Its Modifications in Relation to Enzyme Activity and Biological Functions—A Comprehensive Review. Int. J. Mol. Sci..

[38] Huang Y., Wu Z., Riwanto M., Gao S., Levison B.S., Gu X., Fu X., Wagner M.A., Besler C., Gerstenecker G. (2013). Myeloperoxidase, paraoxonase-1, and HDL form a functional ternary complex. J. Clin. Investig..

[39] Muiz M., Ashok Prabhu K., Durga Rao Y. (2022). Paraoxonase and vitamin D status in subjects with elevated LDL. Biomedicine.

[40] Arzumanian V.A., Kiseleva O.I., Poverennaya E.V. (2021). The Curious Case of the HepG2 Cell Line: 40 Years of Expertise. Int. J. Mol. Sci..

[41] Garcia-Maldonado E., Huber A.D., Chai S.C., Nithianantham S., Li Y., Wu J., Poudel S., Miller D.J., Seetharaman J., Chen T. (2024). Chemical manipulation of an activation/inhibition switch in the nuclear receptor PXR. Nat. Commun..

[42] Gotlieb N., Tachlytski I., Lapidot Y., Sultan M., Safran M., Ben-Ari Z. (2018). Hepatitis B virus downregulates vitamin D receptor levels in hepatoma cell lines, thereby preventing vitamin D-dependent inhibition of viral transcription and production. Mol. Med..

[43] Yang S., Ooka M., Margolis R.J., Xia M. (2023). Liver three-dimensional cellular models for high-throughput chemical testing. Cell Rep. Methods.

[44] Tyakht A.V., Ilina E.N., Alexeev D.G., Ischenko D.S., Gorbachev A.Y., Semashko T.A., Larin A.K., Selezneva O.V., Kostryukova E.S., Karalkin P.A. (2014). RNA-Seq gene expression profiling of HepG2 cells: The influence of experimental factors and comparison with liver tissue. BMC Genom..

[45] Mosmann T. (1983). Rapid colorimetric assay for cellular growth and survival: Application to proliferation and cytotoxicity assays. J. Immunol. Methods.

[46] Livak K.J., Schmittgen T.D. (2001). Analysis of relative gene expression data using real-time quantitative PCR and the 2^−ΔΔCT^ Method. Methods.

[47] Deakin S., Leviev I., Gomaraschi M., Calabresi L., Franceschini G., James R.W. (2002). Enzymatically active paraoxonase-1 is located at the external membrane of producing cells and released by a high affinity, saturable, desorption mechanism. J. Biol. Chem..

[48] Billecke S., Draganov D., Counsell R., Stetson P., Watson C., Hsu C., La Du B.N. (2000). Human serum paraoxonase (PON1) isozymes Q and R hydrolyze lactones and cyclic carbonate esters. Drug Metab. Dispos..

[49] Berman H., Henrick K., Nakamura H. (2003). Announcing the worldwide Protein Data Bank. Nat. Struct. Mol. Biol..

[50] Blaha-Nelson D., Krüger D.M., Szeler K., Ben-David M., Kamerlin S.C.L. (2017). Active Site Hydrophobicity and the Convergent Evolution of Paraoxonase Activity in Structurally Divergent Enzymes: The Case of Serum Paraoxonase 1. J. Am. Chem. Soc..

[51] Molecular Graphics and Analyses Performed with UCSF Chimera, Developed by the Resource for Biocomputing, Visualization, and Informatics at the University of California, San Francisco, with Support from NIH P41-GM103311. https://www.cgl.ucsf.edu/chimera/.

[52] Pettersen E.F., Goddard T.D., Huang C.C., Couch G.S., Greenblatt D.M., Meng E.C., Ferrin T.E. (2004). UCSF Chimera? A visualization system for exploratory research and analysis. J. Comput. Chem..

[53] Šali A., Blundell T.L. (1993). Comparative protein modelling by satisfaction of spatial restraints. J. Mol. Biol..

[54] (2022). Molecular Operating Environment (MOE), 2022.02 Chemical Computing Group ULC,1010 Sherbooke St. West, Suite #910, Montreal, QC, Canada, H3A 2R7. https://www.chemcomp.com/.

[55] Eberhardt J., Santos-Martins D., Tillack A.F., Forli S. (2021). AutoDock Vina 1.2.0: New Docking Methods, Expanded Force Field, and Python Bindings. J. Chem. Inf. Model..

[56] Trott O., Olson A.J. (2010). AutoDock Vina: Improving the speed and accuracy of docking with a new scoring function, efficient optimization, and multithreading. J. Comput. Chem..

[57] Phillips J.C., Braun R., Wang W., Gumbart J., Tajkhorshid E., Villa E., Chipot C., Skeel R.D., Kalé L., Schulten K. (2005). Scalable molecular dynamics with NAMD. J. Comput. Chem..

[58] Dodda L.S., Vilseck J.Z., Tirado-Rives J., Jorgensen W.L. (2017). 1.14*CM1A-LBCC: Localized bond-charge corrected CM1A charges for condensed-phase simulations. J. Phys. Chem. B.

[59] Dodda L.S., de Vaca I.C., Tirado-Rives J., Jorgensen W.L. (2017). LigParGen web server: An automatic OPLS-AA parameter generator for organic ligands. Nucleic Acids Res..

[60] Jorgensen W.L., Tirado-Rives J. (2005). Potential energy functions for atomic-level simulations of water and organic and biomolecular systems. Proc. Natl. Acad. Sci. USA.

[61] Humphrey W., Dalke A., Schulten K. (1996). VMD: Visual molecular dynamics. J. Mol. Graph..

